# A CRISPR RNA Is Closely Related With the Size of the Cascade Nucleoprotein Complex

**DOI:** 10.3389/fmicb.2019.02458

**Published:** 2019-10-29

**Authors:** Do-Heon Gu, Sung Chul Ha, Jeong-Sun Kim

**Affiliations:** ^1^Department of Chemistry, Chonnam National University, Gwangju, South Korea; ^2^Pohang Accelerator Laboratory, Pohang, South Korea

**Keywords:** CRISPR, cascade, Csy3, helical backbone, crRNA

## Abstract

The currently known prokaryotic adaptive immune system against mobile genetic elements is based on clustered regularly interspaced short palindromic repeats (CRISPR). CRISPR-associated (Cas) proteins and the transcribed short CRISPR RNA (crRNA) molecule form a heterologous ribonucleoprotein complex that neutralizes invading foreign nucleic acids, wherein the crRNA molecule base-pairs with the exogenous genetic elements. In the ribonucleoprotein complexes of the type I CRISPR system, a helical backbone of six identical subunits is commonly found. However, it is not clear how this ribonucleoprotein complex is assembled and what is the determinant factor for its size. We elucidated the crystal structure of the Csy3 subunit of the type I-F ribonucleoprotein complex from *Zymomonas mobilis* (ZmCsy3), in which seven ZmCsy3 protomers in the asymmetric unit form a molecular helix that is part of a filamentous structure in the entire crystal system. This ZmCsy3 helical structure is remarkably similar to the crRNA-bound hexameric Csy3 backbone from *Pseudomonas aeruginosa*, with conserved interactions between neighboring subunits. The monomeric ZmCsy3 in solution is transformed into different oligomeric states depending on the added crRNAs. These results suggest that a crRNA and Csy3 subunit play a determinant role in the stepwise formation of the functional Cascade ribonucleoprotein complex and the recruitment of other subunits, and crRNA functions as a molecular ruler for determining the size of the Cascade silencing complex.

## Introduction

Many prokaryotes protect themselves from incoming mobile genetic elements using various defense systems (Westra et al., 2012). The clustered regularly interspaced short palindromic repeat (CRISPR)/CRISPR-associated (Cas) system, which is now classified into six types and has been developed as a gene-editing tool, uses the fragments of past visitors that have been inserted into the CRISPR array for recognition of the re-entering genetic elements (Barrangou et al., 2007; Sapranauskas et al., 2011; Huo et al., 2014, PDB ID 4QQW; Gong et al., 2014, PDB ID 4Q2D; Plagens et al., 2015; Amitai and Sorek, 2016). This CRISPR/Cas system removes invading foreign nucleic acids through three stages: (1) in the acquisition step, a portion of the foreign nucleic acids is selectively included in the CRISPR array; (2) the CRISPR array is transcribed as a single long transcript and processed into several mature small CRISPR RNAs (crRNAs) in the expression step; and (3) the re-entering exogenous nucleic acids are removed by a ribonucleoprotein complex, frequently called the silencing complex, during the interference step (Barrangou et al., 2007; Brouns et al., 2008; Carte et al., 2008; Hale et al., 2009; Garneau et al., 2010; Zhang et al., 2010; Deltcheva et al., 2011; Jore et al., 2011; Gasiunas et al., 2012; Nam et al., 2012, PDB ID 4F3M; Spilman et al., 2013; Staals et al., 2013). The ribonucleoprotein silencing complex, which acts during the interference step, is composed of mature crRNA and Cas protein(s). The mature crRNA in the silencing complex has two sequential motifs of a spacer sequence in the middle and two short repeat sequences at the 3′ and 5′ ends (Brouns et al., 2008). The repeat sequences at both ends have, in many cases, secondary structures of a partial duplex at the 5′-end (5′-handle) and a hairpin structure at the 3′-end (3′-hairpin) (Jore et al., 2011; Jackson et al., 2014, PDB ID 4TVX; Mulepati et al., 2014, PDB ID 4QYZ; Zhao et al., 2014, PDB ID 4U7U). The spacer sequence is related with that of past visitors and forms a hetero-duplex with the nucleic acids of repeat visitors. In contrast to a single protein in the ribonucleoproteins of type II and others (Gasiunas et al., 2012; Zetsche et al., 2015), the type I and type III silencing complexes are composed of a number of hetero polypeptides (Jackson et al., 2014; Mulepati et al., 2014; Zhao et al., 2014; Rouillon et al., 2013; Osawa et al., 2015, PDB ID 3X1L) such that several copies of one component form a protein-helical backbone that provides a binding site for the mature crRNAs and other subunits. These components include Cas7 in the type I system (Jackson et al., 2014; Mulepati et al., 2014; Zhao et al., 2014) and Csm4 and Cmr4 in the type III-A and III-B systems, respectively (Rouillon et al., 2013; Osawa et al., 2015).

In the silencing step of the type I CRISPR systems, the CRISPR-associated complex for antiviral defense (Cascade) silencing complex, composed of Cse1, Cse2, Cas7, Cas5e, and Cas6e, and the Cas3 helicase-nuclease protein work together to form the RNA-mediated DNA loop and to remove the re-entering DNA. The type I-E *Escherichia coli* Cascade, revealed by crystallography (Jackson et al., 2014; Mulepati et al., 2014; Zhao et al., 2014), and the type I-F *Pseudomonas aeruginosa* Cascade, revealed by electron microscopy (EM) (Chowdhury et al., 2017, PDB ID 5uz9; Guo et al., 2017, PUB ID 6NUD), have common structural features and are composed of a protein-helical backbone formed by six copies of Cas7 or Csy3, an embedded crRNA in the helical backbone, and other subunits. However, it is not clear how each component of the silencing complex is stoichiometrically assembled. The mechanism of assembly for the protein-helical Cascade backbone structures of Cas7 or Csy3 remains unknown; specifically, whether their backbone formation is mediated by crRNA or pre-assembled even in the absence of crRNA has not been elucidated.

The microorganism *Zymomonas mobilis* ZM4 has three types of CRISPR systems (Seo et al., 2005). Among them, four genes of Csy1, -2, -3, and -4 (ZmCsy1-4) appear to form a silencing Cascade complex in the type I-F CRISPR systems. Based on the *P. aeruginosa* Cascade complex with a protein-helical backbone of six Csy3s, ZmCsy3 is predicted to form the molecular-helical backbone of the *Z. mobilis* Cascade complex. In order to provide a clue for the assembly mechanism of the Cascade silencing complex of the type I CRISPR/Cas system, the crystal structure of ZmCsy3 was determined. Interestingly, this protein forms a filament even in the absence of crRNA, which is same as the Csy3 backbone structure observed in the crRNA-bound type I-F Cascade of *P. aeruginosa* (Chowdhury et al., 2017; Guo et al., 2017). The monomeric ZmCsy3 protein forms different oligomers in proportion to the added crRNAs of different lengths in solution. These results, together with the reported data, suggest that crRNA may play a major role in assembly of the Cascade complex and determine its size.

## Materials and Methods

### Construction of a Recombinant Plasmid and Purification of ZmCsy3

The Csy3 gene (ZMO0684, Met1-Ser346) has 1041 nucleotides, which encode a polypeptide of 346 residues. The target gene was amplified to construct a recombinant plasmid from *Z. mobilis* ZM4 genomic DNA by polymerase chain reaction (PCR) using primers with two restriction enzymes. The amplified PCR product was treated with the restriction enzymes *Nco*I (New England Biolabs, Beverley, MA, United States) and *Xho*I (New England Biolabs, Beverley, MA, United States) and was inserted into the vector pHIS2, a derivative of pET21a (Novagen, Madison, WI, United States) that was designed to translate the protein in a fused state with a 6xHis and tobacco etch virus (TEV) protease cleavage sequence at the N-terminus. The constructed recombinant plasmid was transformed into *Escherichia coli* BL21Star(DE3), which was grown in Luria-Bertani (LB) media supplemented with 50 mg⋅mL^–1^ ampicillin at 291 K. When the optical density at 600 nm reached 0.3, the fusion protein was expressed by adding 1.0 mM isopropyl β-D-1-thiogalactopyranoside (IPTG) into the culture media followed by incubation for an additional 18 h at 291 K. The culture was harvested by centrifugation at 5,000 g at 277 K. The cell pellet was resuspended in an ice-cold buffer A consisting of 20 mM Tris–HCl pH 7.5, 10 mM β-mercaptoethanol, 500 mM NaCl, and 5% (v/v) glycerol, and then disrupted by ultrasonication. Cell debris was removed by centrifugation at 11,000 *g* for 30 min. The expressed ZmCsy3 fusion protein was initially bound by His-binding agarose resin (ELPIS, Daejeon, South Korea) and the bound protein was eluted by a 500 mM imidazole gradient in buffer A. The eluted ZmCsy3 protein was incubated with the TEV protease to cleave the 6xHis at the N-terminus and simultaneously dialyzed to remove the salt. After dialysis, the added TEV protein was removed by reloading the protein on the His-binding agarose resin (ELPIS, Daejeon, South Korea). For further purification, anion exchange chromatography was performed using the 5 ml HiTrap^TM^ Q column (GE Healthcare, Uppsala, Sweden). The bound protein was released by a linear gradient from 0 to 1 M NaCl in buffer A. The final purified protein was concentrated up to 13 mg⋅ml^–1^ in a buffer consisting of 20 mM Tris–HCl pH 7.5 and 200 mM NaCl for crystallization. The protein concentration of the purified ZmCsy3 was determined by taking into consideration of its extinction coefficient (1.035 M^–1^⋅cm^–1^) at 280 nm.

### Crystallization and Structure Determination

The purified ZmCsy3 was crystallized using the sitting-drop vapor-diffusion method in 96-well MRC Crystallization Plates (Molecular Dimension, Maumee, OH, United States). Initial crystallization conditions were screened with the sparse-matrix method (Jancarik and Kim, 1991) using commercially available crystallization kits from Hampton Research (Aliso Viejo, CA, United States) and Rigaku Reagents (Bainbridge Island, WA, United States). The protein solution (1 μl) at a concentration of 13 mg⋅ml^–1^ was mixed with equal volumes of the screen reagents and the resulting drops were equilibrated against 70 μl reservoir solution at 22°C. The initial conditions were optimized with the hanging-drop vapor-diffusion method using the VDX plate (Hampton Research). The best crystals were grown in the presence of 0.1 M Bis-Tris pH 6.5 and 2.0 M ammonium sulfate. For data collection, a single crystal was cryo-protected with the reservoir solution supplemented with 20% xylitol and flash-frozen directly in a −173°C nitrogen stream using a nylon loop. A data set consisting of 360 frames of X-ray diffraction data were collected on an ADSC Q270 CCD detector at beamline 7A of the Pohang Light Source II (PLS II) at Pohang Accelerator Laboratory (PAL, Pohang, South Korea) using 0.5° oscillations at a wavelength of 0.9793 Å. The data were indexed and scaled with the HKL-2000 software package (Otwinowski and Minor, 1997). The crystallographic data statistics are summarized in Table 1. The structure was determined with the molecular replacement method in the PHENIX package (Adams et al., 2010; phaser) using the Csy3 molecule (PDB ID 5UZ9; Chowdhury et al., 2017) of *P. aeruginosa* as a search model. The seven models identified were refined with the PHENIX package and the resulting electron density was used for manual rebuilding using Coot (Emsley and Cowtan, 2004). The final model was obtained after iterative manual model building and refinement with Coot and the PHENIX package, respectively. The statistics of refinement are shown in Table 1. Molecular model figures were prepared using the PyMol Molecular graphics program (Schrödinger, LLC). The atomic coordinates and structure factors were deposited in the Protein Data Bank using accession code 6KQR^1^.

**TABLE 1:** Data collection and refinement statistics for ZmCsy3.

**Statistics**	**Se-Met ZmCsy3**
**Data collection**	
Space group	P1
Cell dimensions (Å)	
a, b, c (Å)	82.18, 116.16, 115.94
α, β, γ (°)	99.36, 103.12, 103.3590
Resolution (Å)	30.0–2.9 (2.95–2.9)
R_merge_^a^(%)	6.7 (46.9)
CC_1__/__2_^b^	0.942 (0.745)
I/σ (I)	16.0 (2.0)
Completeness (%)	98.4 (97.9)
Redundancy	1.9 (1.9)
Wilson B factor (Å^2^)	56.92
**Structure refinement**	
Resolution (Å)	30.0 – 2.9
Reflections, total/test set	86047/3438
R_work_^b^/R_free_	19.2 (32.5)/25.9 (47.1)
No. atoms, protein/water	18907/763
R.m.s. deviation	
Bond lengths (Å)	0.008
Angles (°)	1.015
Average B-factor (Å^2^)	
Protein/water	81.5/71.2
Ramachandran plot (%)	
Favored region	91.47
Allowed region	6.42

*The numbers in parentheses are statistics from the highest resolution shell. *^*a*^*R**_*merge*_ = Σ |*I*_*obs*_ –*I*_*avg*_|/*I*_*obs*_, where *I*_*obs*_ is the observed intensity of individual reflection and *I*_*avg*_ is the average over symmetry equivalents. ^*b*^Karplus and Diederichs (2012). *^*c*^*R**_*work*_ = Σ || *F*_*o*_|– |*F*_*c*_||/Σ |*F*_*o*_|, where |*F*_*o*_| and |*F*_*c*_| are the observed and calculated structure factor amplitudes, respectively. *R*_*free*_ was calculated with 4% of the data.*

### Analytic Size Exclusion Chromatography

The crRNA of *P. aeruginosa* (CUAAGAAAUUCACGGCGG GCUUGAUGUCCGCGUCUACCUGGUUCA CUGCCGUAU AGGCAG, the spacer sequences underlined) and its short version (UCACGGCGGGCUUGAUGUCCGCGUCUACCU) containing a 30-nucleotide spacer (from +2 to +31 position of crRNA) were synthesized (Bioneer, South Korea). Each crRNA was mixed with ZmCsy3 at a protein to crRNA ratio of 11 to 1 and 5 to 1, respectively, and incubated for more than 2 h at 22°C. The mixtures were analyzed with a Superdex 200 10/30 column (GE Healthcare) equilibrated with 20 mM Tris pH 7.5, 150 mM NaCl, and 5% glycerol. The molecular weight of the eluates was estimated based on the positions of known molecular-weight proteins (ferritin, 440 kDa; aldolase, 158 kDa; ovalbumin, 44 kDa; carbonic anhydrase, 29 kDa; ribonuclease A, 13.7 kDa).

## Results

### Structural Features of *Zymomonas* Csy3

**FIGURE 1:**
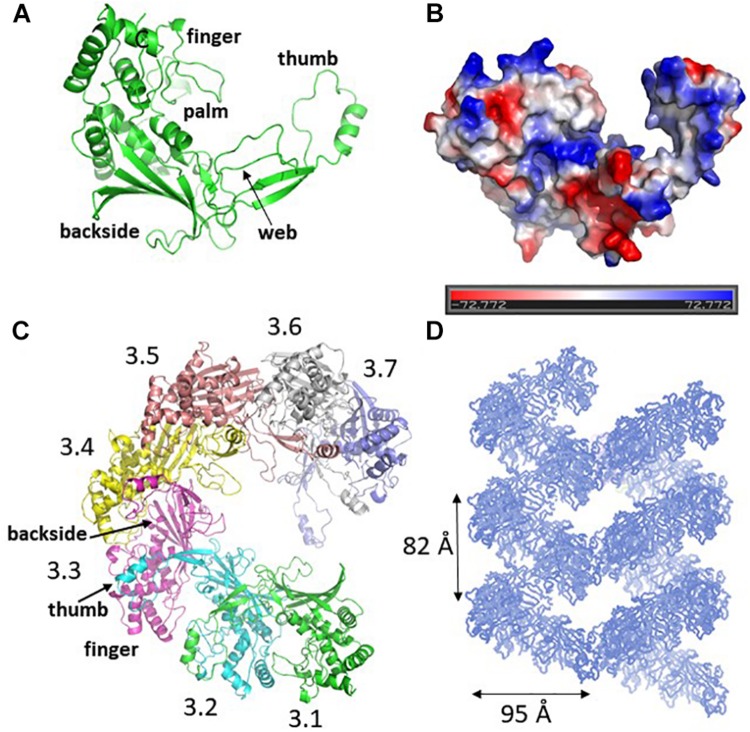
Crystal structure of ZmCsy3. **(A)** The monomeric structure of ZmCsy3 is presented as a ribbon diagram. Each structural motif is indicated. **(B)** Surface potential map of the ZmCsy3 molecule in **(A)**. The surface electrostatic potential was calculated using the APBS server (http://www.poissonboltzmann.org/) and is visualized as a color ramp from blue (positive) to red (negative). This figure was rotated with respect to **(A)**. **(C)** The molecular helix formed by seven Csy3 molecules in the asymmetric unit. Each molecule is differentiated by colors and labeled from 3.1 to 3.7 to indicate the first to 7th Csy3 molecules. **(D)** The filamentous structures formed by Csy3 molecules. The symmetry-related molecules are displayed with coils.

The crystal structure of Csy3 from *Z. mobilis* ZM4 (ZmCsy3, PDB ID 6KQR) was determined by the molecular replacement method at a resolution of 2.9 Å using the Csy3 component structure of the *P. aeruginosa* Cascade complex (PDB IDs 5UZ9 and 6B44) as a search model (Chowdhury et al., 2017; Guo et al., 2017). There are seven ZmCsy3 molecules (ZmCsy3.1–ZmCsy3.7) in the asymmetric unit, and their structures superimpose very well onto each other, with a root-mean-square-deviation (rmsd) value of less than 0.2 Å. The refined structure lacks the first 9 residues at the N-terminus (Met1-Thr9). The monomeric ZmCsy3 structure (Figure 1A) is composed of ten α-helices (α1-α10) and eight β-strands (β1–β8) and forms a right-handed glove that is composed of a finger of several α-helices, a palm consisting of a β-sheet of 6 β-strands with an extended region (web), a thumb composed of a two-stranded β-sheet and an α-helix, and the backside of glove. The surface potential map reveals a positively charged surface patch within the glove and the inner sides of a finger and the thumb, and negatively charged surface patches on the backside of the glove and the outside of a finger and a thumb (Figure 1B).

The seven ZmCsy3 molecules in the asymmetric unit form an inter-molecular helical structure (Figure 1C). At the inter-subunit interface, a thumb of one subunit interacts with a finger of the neighboring subunit, and the β-sheet of the five β-strands on the backside of one subunit comes into contact with those of the neighboring subunit(s) through many polar and hydrophilic interactions. In this oligomeric structure, the concave palms are twisted within a single molecule and form a continuous hollow cleft along the molecular helix, together with the extended regions (webs) and several loops in the fingers, and expose a long positively charged cleft to the solvent.

The inter-molecular helix formed by seven ZmCsy3 molecules is part of a long superhelical filament structure in the entire crystal system, where seven molecules form a single turn with a diameter of ∼95 Å and a length of ∼82 Å (Figure 1D). Since this molecular-helical structure is formed in the absence of crRNA in the crystalline state, there is a possibility that ZmCsy3 can be aggregated at high protein concentrations to form a molecular helical backbone of the Cascade effector complex without crRNA.

### Comparison With Other Cascade Backbones

**FIGURE 2:**
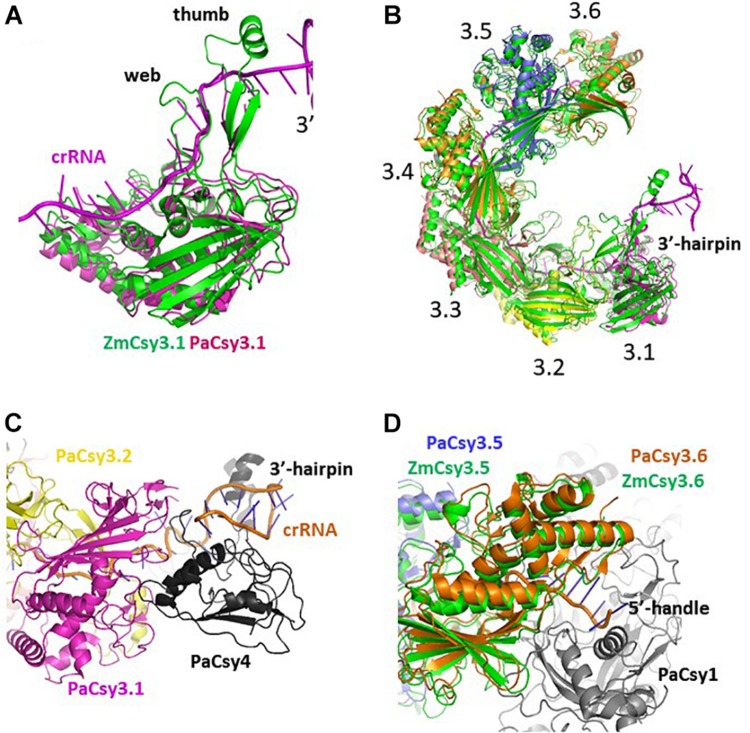
Comparison of Csy3 subunits in the apo- and crRNA-bound Cascade backbone. **(A)** Superimposed structure of the two monomeric Csy3 structures from *Z. mobilis* and *P. aeruginosa*. The two Csy3 structures are differentiated with colors and the crRNA bound to the PaCsy3 is displayed with a ribbon diagram. **(B)** The superimposed hexameric Csy Cascade backbone. The ZmCsy3 molecules are displayed in green, while the PaCsy3 molecules are shown with alternating colors. The crRNA bound to the PaCsy3 backbone is displayed as a ribbon. Each Csy3 molecule is labeled from 3.1 at the 3′-hairpin to 3.6 at the 5′-handle. **(C)** A close-up view of the subunit interaction at the 3′-hairpin region. The 3′-hairpin-interacting PaCsy4 subunit in the *P. aeruginosa* Cascade complex is displayed as a black ribbon. **(D)** Close-up view of the subunit interaction at the 5′-handle region. The 5′-handle-interacting PaCsy1 subunit in the *P. aeruginosa* Cascade complex is displayed as a black ribbon.

There are two Cys3 proteins, including the ZmCsy3 studied here, whose structures have been reported (Figure 2; Chowdhury et al., 2017; Guo et al., 2017). ZmCsy3 and *P. aeruginosa* Csy3 (PaCsy3) have a high sequence identity of ∼60% with some gaps in three regions of the aligned sequences: one is located in the backside of the glove motif and the other two regions are found in the extended loop of the palm motif in the monomeric Csy3 structures (Supplementary Figure S1). The revealed monomeric ZmCsy3 structure superimposes well onto the PaCsy3 structure with rmsd values of less than 1.5 Å among the aligned 325 Ca atoms out of ∼340 residues (Figure 2A). The largest deviations are found in the thumb and the web extended from the palm. Since these deviated regions interact with the bound crRNA in the PaCsy3 of the *Pseudomonas* Cascade-crRNA complex (Chowdhury et al., 2017; Guo et al., 2017), it is likely that these flexible regions re-orient according to the accessing crRNA.

In the known type I CRISPR Cascade silencing complexes (Jackson et al., 2014; Mulepati et al., 2014; Zhao et al., 2014; Hochstrasser et al., 2016; Chowdhury et al., 2017; Guo et al., 2017; Xiao et al., 2017, PDB ID 5U07; Xiao et al., 2018, PDB ID 6C66), six Cas7 or Csy3 molecules are aggregated into a molecular helical structure, which serves as a docking site for crRNA and other Cascade components. Interestingly, the elucidated molecular helix formed by seven ZmCsy3s without crRNA is strikingly similar to the helical backbone of the hexameric PaCsy3 and EcCas7 (Figure 2). The hexameric molecular-helix formed by ZmCsy3s, except for one ZmCsy3 subunit at either end in the apo-backbone structure, superimposes well onto the PaCsy3 helix backbone in the holo-Cascade structure, with rmsd values of less than 2.51 Å among the aligned 1912 Ca atoms, and only a negligible difference at the crRNA binding regions (Figure 2B). These structural features strongly indicate that the backbone-forming proteins of the type I Cascade complex might self-assemble before binding crRNA and other subunits.

### The Monomeric ZmCsy3 Can Form Oligomeric Structures With crRNA in Solution

Because the observed molecular helix formed by six ZmCsy3 subunits in the crystalline state closely resembles the molecular helix of PaCsy3 in the holo-Cascade complex (Figure 2), we performed gel-filtration analysis to verify the oligomeric state of ZmCsy3 in solution using the purified ZmCsy3 protein in the presence and absence of various crRNAs.

**FIGURE 3:**
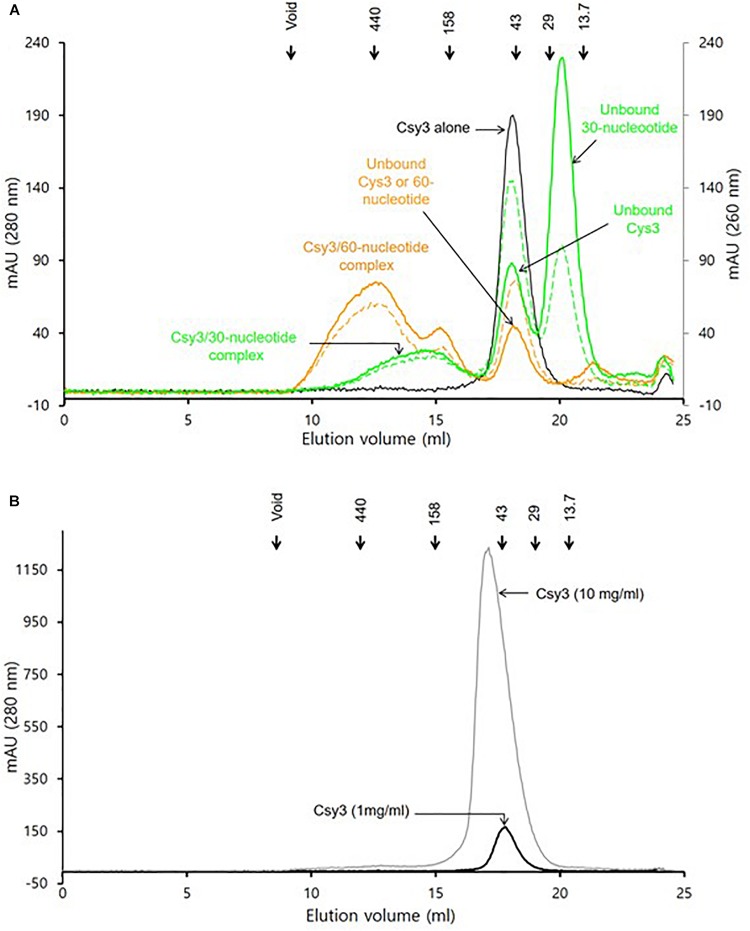
Analysis of the oligomeric state of ZmCsy3 with and without crRNA. **(A)** The size distribution of ZmCsy3 with 60-nucleotide crRNAs (orange line, 260 nm: orange dash, 280 nm) or 30-nucleotide crRNAs (green line, 260 nm: green dash, 280 nm) and ZmCsy3 alone (black line, 280 nm) were checked using an analytic size exclusion column. **(B)** The concentration dependent oligomeric state of ZmCsy3 was checked at the concentration (black line, 1 mg/ml) used for the complex formation with crRNA and the 10-fold excess concentration (gray line, 10 mg/ml). The known standard molecular weights in kDa are marked in the elution profile.

The gel filtration assays revealed that the purified ZmCsy3 protein of 1 mg/ml migrates as a monomer in the absence of crRNA (Figure 3A), while a ZmCsy3 protein of 10 mg/ml eluted a little earlier than that of 1 mg/ml (Figure 3B). In contrast, recombinant ZmCsy3 migrated in oligomeric forms when a 60-nucleotide crRNA derived from the CRISPR array of *P. aeruginosa*, which is expected to bind with six Csy3 molecules, was added into the ZmCsy3 protein solution. Interestingly, adding a 30-nucleotide spacer ranging from the +2 to +31 position of a 60-nucleotide crRNA, which is supposed to bind with four ZmCsy3 subunits in a sequence-independent manner, also transformed the monomeric ZmCsy3 into an oligomeric protein. However, the assembled size with the 30-nucleotide spacer crRNA was smaller than with the major oligomer formed by the 60-nucleotide crRNA, indicating that the spacer length of crRNA may be related to the number of recruited ZmCsy3 molecules.

## Discussion

Structural studies of the type I and III silencing effector complexes of the CRISPR systems have often revealed a helical backbone of one subunit that is flanked by different subunits at both ends and provides binding sites for crRNA and other subunits (Jackson et al., 2014; Mulepati et al., 2014; Zhao et al., 2014; Chowdhury et al., 2017; Guo et al., 2017, 2019; Xiao et al., 2017, 2018, PUB ID 6NUD; Jia et al., 2019, PDB ID 6MUR; You et al., 2019, PDB ID 6IFN). In the type I-F system, several Csy genes comprise a silencing Cascade complex with the Csy3 protein as a backbone-forming molecule (Chowdhury et al., 2017; Guo et al., 2017).

Our analysis using gel filtration assay indicated that ZmCsy3 migrates as a monomeric protein at low concentrations (Figure 3A) and does not form a higher oligomeric state at a concentration of 10 mg/ml (Figure 3B). Nonetheless, there is a high possibility that at high protein concentrations, such as when it is the crystalline state, the Csy3 subunit itself can form an oligomeric structure, which would be very similar to that observed in our current ZmCsy3 oligomeric structure (Figure 2) as well as the Cas7 backbone of the crRNA-bound Cascade complex (Jackson et al., 2014; Mulepati et al., 2014; Zhao et al., 2014; Xiao et al., 2017, 2018). Interestingly, the crRNA-deficient Cmr4 subunit also form a filament structure consisting of eight molecules per turn of 195 Å in the type III-B effector complex (Zhu and Ye, 2015, PDB ID 4WNZ). Even though this Cmr4 filament is similar to that of ZmCsy3, they are rather different in that the ZmCsy3 apo-filament has seven molecules per a single turn with a much shorter distance of 82 Å than that of Cmr4.

The molecular helical backbones formed by Csm3 subunits in type III-A effector complexes have various copies of the Csm3 subunit (Guo et al., 2019; Jia et al., 2019; You et al., 2019). While two or three Csm3 subunits are observed with a short 36-nucleotide crRNA (Jia et al., 2019; You et al., 2019), five Csm3 copies are observed with a 72-nucleotide crRNA (Guo et al., 2019). Similarly, different numbers of Cas7 subunits have been reported depending on the length of the added crRNA; for example, three Cas7fv in the type I-F in *Shewanella putrefaciens* with a 43-nucleotide crRNA (Gleditzsch et al., 2016) and three to six Cas7 molecules in *E. coli* and *T. fusca* with altered crRNAs (Jackson et al., 2014; Mulepati et al., 2014; Zhao et al., 2014; Kuznedelov et al., 2016; Luo et al., 2016; Xiao et al., 2017). The molecular helical-forming Cmr4 subunits in the type III-B systems are also found in different numbers (Osawa et al., 2015; Zhu and Ye, 2015). Here, our oligomerization assays with the ZmCsy3 protein, which exists as a monomeric protein at low concentrations, have shown aggregated ZmCsy3 proteins of different sizes in proportion to the lengths of the added crRNAs (Figure 3). These features, along with the reported data, strongly suggest that the length of crRNA might be a critical factor for determining the molecular helical backbone size of the silencing complex.

The Cascade silencing complex has a molecular helical backbone of six Cas7 molecules that is flanked by Cas6e and Cas5e at both end, and the former and the latter interact with the 3′-hairpin and the 5′-handle regions of the crRNA, respectively (Jackson et al., 2014; Mulepati et al., 2014; Zhao et al., 2014; Hochstrasser et al., 2016; Xiao et al., 2017, 2018). In this complex structure, the 3′-hairpin structure of crRNA is buried within the protein Cas6e that interacts with Cas7 at one end of the molecular backbone of the Cascade complex, while the 5′-handle region of crRNA is buried within the Cas5e subunit. As mentioned above, crRNA sequences are divided into spacer and repeat sequences, where the relatively well conserved repeat sequence at both ends of a single mature crRNA forms rigid secondary structures, and the variable and elongated spacer sequence in the middle of the crRNA sequence is a multiple of six nucleotides and forms a duplex with exogenous genetic elements through base-pairing. These interactions between the crRNA and the Cascade subunits are also conserved in the type I-F system (Rouillon et al., 2013; Chowdhury et al., 2017; Guo et al., 2017) as well as the type III effector complexes (Osawa et al., 2015). Therefore, the partial secondary structures formed at both ends of crRNA are likely to interfere with the regular interactions between the two molecular helix-forming subunits at the proximal sites and to induce favorable interactions with other Cascade subunits. Our gel filtration assays, conducted with crRNAs that have the same conserved 5′-handle and 3′-hairpin regions but different lengths of spacer sequences, have shown that a small crRNA forms a Csy3 oligomeric protein that is smaller than that formed by a longer crRNA (Figure 3). Taken together, these results suggest that a crRNA with the 3′-hairpin and 5′-handle can be a ruler to guide how many molecules should be put together to form the Cascade backbone, and can play a key role in coordinating the assembly of Cascade subunits for the formation of the catalytically active silencing complex, which determines the size of the Cascade, in addition to its well-known function of recognizing exogenous nucleic acids through base-paring between the crRNA and the exogenous nucleic acids.

## Data Availability Statement

The datasets generated for this study can be found in the Protein Data Bank 6KQR.

## Author Contributions

J-SK designed the study. All authors performed the experiments, analyzed the data, and wrote the manuscript.

## Conflict of Interest

The authors declare that the research was conducted in the absence of any commercial or financial relationships that could be construed as a potential conflict of interest.

## Footnotes

1 http://www.rcsb.org/pdb
