# Supplementary material for: A CRISPR RNA Is Closely Related With the Size of the Cascade Nucleoprotein Complex
Source: Front Microbiol. 2019 Oct 29;10:2458. doi: 10.3389/fmicb.2019.02458 (PMC6828817; doi:10.3389/fmicb.2019.02458)
Supplement: Supplementary file 1 [file Data_Sheet_1.docx]

**Suppl. Image**

**A CRISPR RNA is closely related with the size of the Cascade nucleoprotein complex**

Do-Heon Gu^1^, Sung Chul Ha^2,*^, and Jeong-Sun Kim^1,*^

^1^ Department of Chemistry, Chonnam National University, Gwangju 61186, Republic of Korea

^2^ Pohang Accelerator Laboratory, Pohang, Gyeongbuk 37673, Republic of Korea

Gu and Ha equally contributed.

**Supplementary figure S1**


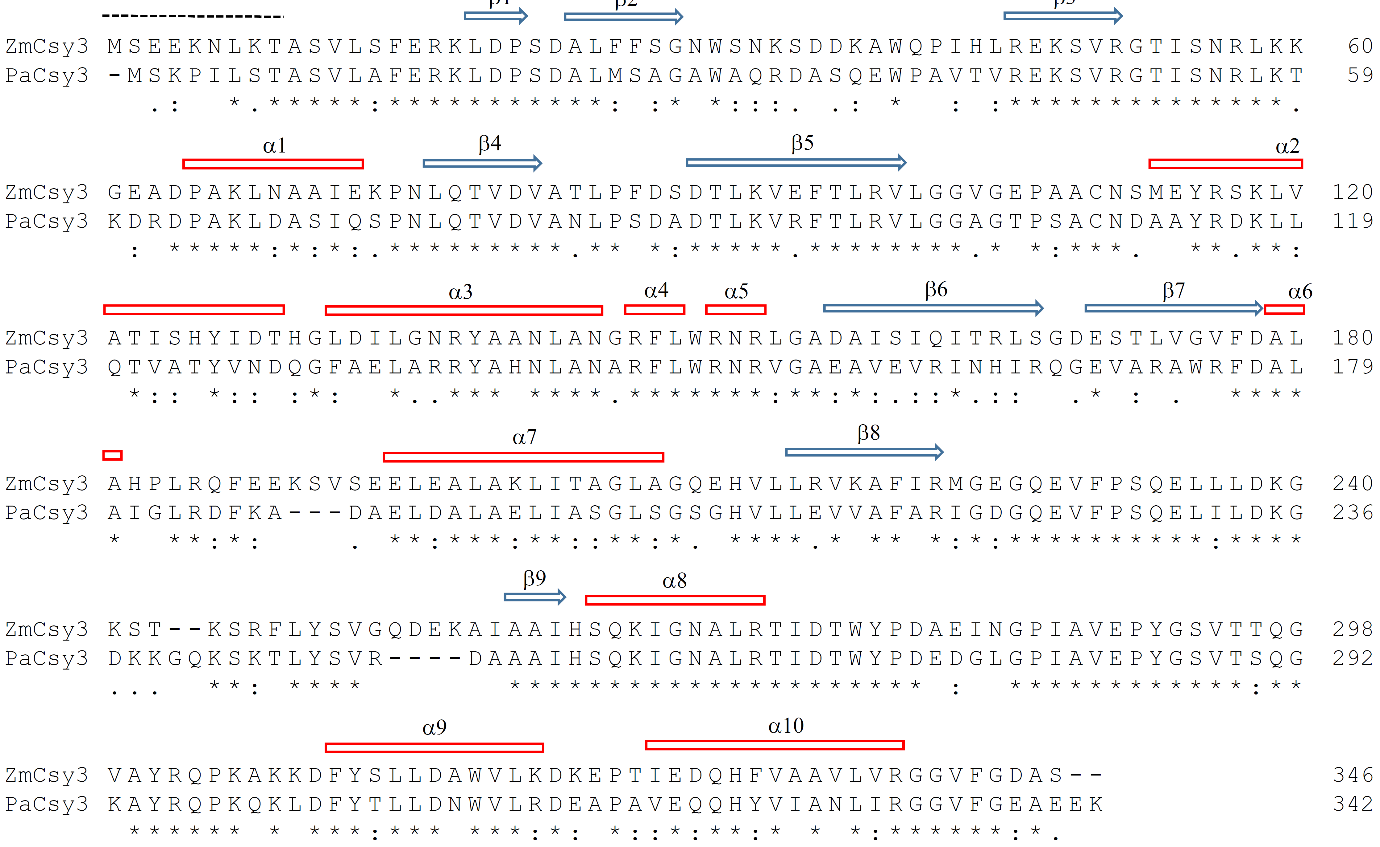


Sequence alignment of two Csy3 proteins from different microorganisms.

The α-helices and β-strands are depicted as rectangular boxes and arrows, respectively, above the aligned sequences. The dotted lines indicate untraced regions. ZmCsy3, Csy3 from *Zymomonas mobilis*; PaCsy3, *Pseudomonas aeruginosa*.
